# Measurement Tools and Utility of Hair Analysis for Screening Adherence to Antihypertensive Medication

**DOI:** 10.5334/gh.1191

**Published:** 2023-03-22

**Authors:** Jyoti R. Sharma, Phiwayinkosi V. Dludla, Girish Dwivedi, Rabia Johnson

**Affiliations:** 1 Biomedical Research and Innovation Platform, South African Medical Research Council, Tygerberg 7505, South Africa; 2 Medical School, University of Western Australia, Harry Perkins Institute of Medical Sciences, Fiona Stanley Hospital, Verdun Street, Nedlands WA, 6009, Australia; 3 Centre for Cardio-Metabolic Research in Africa, Division of Medical Physiology, Faculty of Medicine and Health Sciences, Stellenbosch University, Tygerberg 7505, South Africa

**Keywords:** hypertension, drug/treatment adherence, compliance, antihypertensive medication

## Abstract

Poor adherence to the prescribed antihypertensive therapy is an understated public health problem and is one of the main causes of the high prevalence of uncontrolled hypertension in sub-Saharan Africa. Medication adherence is vital for the effectiveness of antihypertensive treatment and is key to ameliorating the clinical outcomes in hypertensive patients. However, it has often been ignored because the current methods used to assess medication adherence are not reliable, limiting their utilization in clinical practice. Therefore, the identification of the most accurate and clinically feasible method for measuring medication adherence is critical for tailoring effective strategies to improve medication adherence and consequently achieve blood pressure goals. This review not only explores various available methods for estimating medication adherence but also proposes therapeutic drug monitoring in hair for the measurement of medication adherence to the antihypertensive medication period.

## 1. Introduction

The prevalence of hypertension is projected to rise by 60% in the next decade, with a disproportionately higher prevalence rate in the African region [1]. In 2008, 46% of adults aged 25 or over were hypertensive [2], and 75% or more of hypertensive patients are not able to achieve their target blood pressure (BP) goals [3,4,5,6]. Various factors, such as lack of proper treatment (cost and unavailability of medicines), clinical inertia (reluctance to intensify medication), and interruption of medication [7,8,9], have been evaluated to investigate their role in BP control. It has been postulated in several studies that poor adherence to medications is one of the major determinants of the high prevalence of uncontrolled hypertension [6,8,10]. Poor or nonadherence to the prescribed therapy may occur anytime while taking medication. Of course, a patient may decide anytime to stop his/her medication at the start or later due to certain beliefs, acute side effects, forgetfulness, carelessness, or for other personal or socioeconomic reasons [11]. Among patient-related factors, failure to accept the disease diagnosis is a major obstacle to medication adherence. If patients consider that prescribed medications will not be effective in controlling BP or will result in severe side effects, then poor adherence is observed [12,13]. It has recently been reported that many factors, including lack of knowledge, negative attitudes, and negative beliefs, resulting in poor quality of life contribute to poor adherence to medication in low- and middle-income countries [14].

Monitoring antihypertensive therapy adherence is critical for the achievement of optimal BP control, prevention of consequent cardiovascular events, and the identification of truly resistant patients [6,7,8]. However, measuring adherence to the treatment has often been ignored because of the subjective nature of the methods utilized to assess nonadherence and their limited use in clinical practices. Adherence to the medication can save billions, not only in terms of direct health care costs but also in indirect costs related to loss of economic productivity [15,16]. Therefore, the accurate assessment of nonadherence is pivotal in planning interventions to control hypertension, prevent complication, and limit adverse effects of the treatment [17].

To achieve this objective, it is critical to identify the most accurate and clinically feasible adherence assessment method that may add to early behavioral intervention, prevent unnecessary diagnostic testing, and perhaps also limit the use of expansive BP-lowering methods. There are several methods to measure drug adherence; however, each method has advantages and disadvantages, and not a single method can be considered as a gold standard. To overcome the limitations of the most available methods, hair analysis has been proposed for the measurement of treatment adherence among hypertensive patients. This method has previously been used in forensic studies to unveil a person’s drug abuse history and in the investigation of drug-facilitated crime cases [18,19,20,21]. Further, drug deposition in hair has also been utilized in various clinical settings to examine the accumulation of drugs and their metabolites for the assessment of adherence to antiretroviral therapy [22,23]. However, very limited literature is available on utilizing hair analysis for the assessment of adherence to hypertension treatment [24].

**Figure 1:**
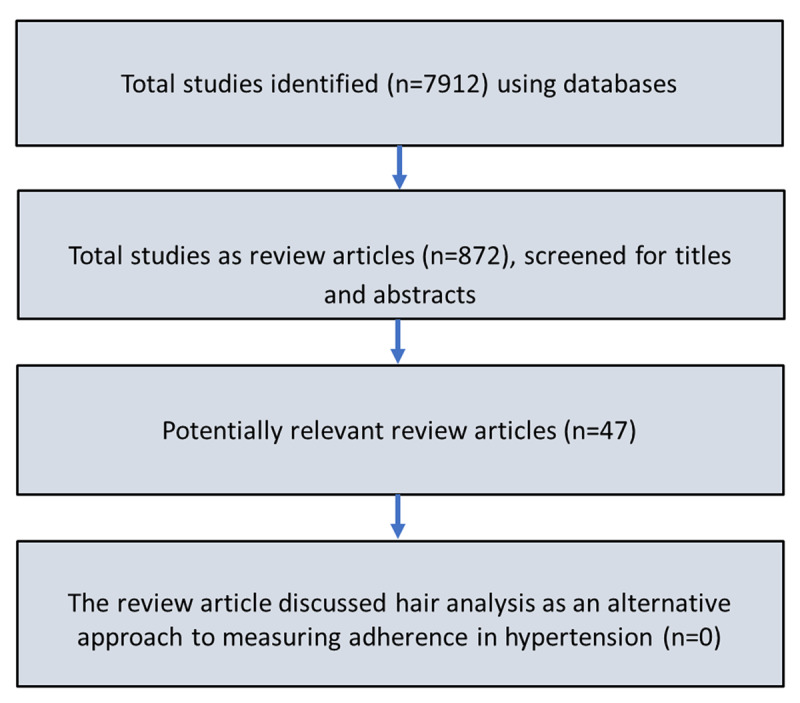
Flow chart of literature search.

In this review, we will briefly discuss various available methods and propose hair analysis as an alternative method for the identification of nonadherence to antihypertensive medications. The literature search was conducted on various databases, including Medline, Google Scholar, Scopus, and African Journals to identify review articles published from 1977 to 2022 that discuss the methodology for the assessment of medication adherence in the adult hypertensive population. A MeSH search using the keywords “methods,” “medication adherence/drugs adherence/drug compliance,” “hypertension,” “treatment-resistant hypertension,” and “uncontrolled hypertension” retrieved 7,912 research articles (Figure 1). However, 872 were identified as review articles and were screened for titles and abstracts; the further search was narrowed down to only 47 review articles that discussed various methods of revealing and measuring medication adherence, and none of the reviews mentioned investigating drug levels in hair as an alternative approach for the measurement of adherence among hypertensive individuals. Therefore, to fill this gap, the current review has not only discussed available methods to measure adherence but also proposed to do hair analysis for therapeutic drug monitoring (TDM) in treatment-resistant hypertension.

This review summarizes currently available methods for the measurement of adherence along with their advantages and disadvantages and then discusses the methods being used for measuring medication adherence on the African continent. Later, an alternative method of TDM highlighting hair analysis for the estimation of therapeutic drug adherence is explored that may enhance the identification of drug nonadherence in low-resource settings.

## 2. Measuring Adherence: Methods with Applicability and Challenges

Measurement of medication adherence is quite challenging since the parameters of accepted adherence methods should be clearly described and appropriate to the individual situations [25]. The available methods for measuring medication adherence in hypertensive subjects (direct and indirect methods) along with the pitfalls in their applicability in clinical settings are summarized in Tables 1 and 2 and are described below.

**Table 1:** Descriptive information of different questionnaires for the assessment of medication adherence.


QUESTIONNAIRE	BRIEF DESCRIPTION AND FUNCTION	RELIABILITY	LIMITATIONS	REFERENCES

**Morisky, Green, Levine (MGL) scale**	Four-item Morisky scale Short and concise Assesses frequency of missing medicine because of forgetfulness Barriers to treatment adherence Symptom severity	Validated assessment tool for nonadherence to medication Cronbach’s α = 0.61 Sensitivity and specificity value 81% and 44%, respectively	Overestimation	[33,37,64]

**Morisky Medication Adherence Scale (MMAS-8)**	Eight-item Morisky modified adherence scale Assesses nonadherence due to feelings of pressure or reasons other than forgetfulness Better psychometric problems assessment	More popular than the four-item Morisky scale Cronbach’s α = 0.68 Has been used in clinical settings Translated and validated in various countries Sensitivity and specificity values of 93% and 53%, respectively	Recall bias Does not comprehensively assess the predictors of nonadherence Moderate performance in the identification of patients without medication adherence problems	[46,51,65,66]

**The Hill-Bone Compliance to High Blood Pressure Scale (HBCHTS)**	Assesses patient behaviors for three important aspects: Decreased sodium intake Adherence to the appointment Taking of medication	Validated for clinical settings in South Africa Validated focusing on cultural sensitivity and appropriateness for low literacy More detailed info on compliance behavior Cronbach’s α = 0.43–0.84	Does not provide a cutoff point for optimal or poor adherence Limited clinical outcome correlation with adherence	[38,67,68,69,70]

**Turkish HBTS**	Used in primary settings in Turkey Considers unintentional and intentional medication nonadherence Prevents patient’s boredom and loss of attention	Good construct validity and reliability Validated for clinical settings Validated for primary settings in Turkey Cronbach’s α = 0.83 for medication compliance, 0.62 for salt intake items, and 0.72 for whole scale of HBTS	Limited to primary settings in Turkey	[60]

**Brief Medication Questionnaire (BMQ)**	Self-report tool for screening adherence and barriers to adherence Based on a 5-item regimen scale Focuses on a drug regimen Measures barriers to adherence	Sensitivity level of 80% and specificity level of 100% Cronbach’s α = 0.6	Needs clinical studies to validate the utility in primary care settings	[41,68]

**Adherence self-report questionnaire**	Based on six descriptions associated with six levels of adherence Able to assess adherence to medication	Sensitivity values of 1442% High specificity values (90%–93%)	Less ability to detect true nonadherence Possible role of Hawthrone effect (change in patient’s behavior due to being monitoried) cannot be ignored Needs to validate the findings in patients with low adherence to medication Short monitoring period (33 days)	[39,71]

**Stages of change (SOC) for adherence measure**	Predicts behavior changes in patients Two-item measure of SOC for adherence to medication regimens	Construct validity confirmed with the association of SOC with previously measured levels of adherence	Needs to be validated in clinical settings Generic stage-tailored approach Chances of social desirability bias	[40]


**Table 2:** Methods of measuring medication adherence in the management of hypertension.


METHODS	IN USE GLOBALLY	UTILIZED IN AFRICA	ADVANTAGES	DISADVANTAGES	REFERENCES

INDIRECT METHODS					

Patient interviews	Yes	Yes	Easy Cheapest	Greatly biased Often provides limited and irrelevant information Overestimation of adherence	[25,26,27,43] [28,29,30]

Questionnaires	Yes	Yes	Easy Inexpensive Ability to provide reasonable information	Provides overestimated information about adherence Recall bias Can be challenging and time-consuming for illiterate patients	[36,44,45,46,48,49,50,54,72]

Pill counting	Yes	Yes	Cost-effective Simplest Can be used in various formulations	Cumbersome for patient and assessor Time-consuming Does not ascertain time, consumption, dose, or frequency of medication Inaccurate Underestimation due to early refill	[73,74,75,76,77,78,79,80,81]

Electronic monitoring	Yes	Yes	Highly accurate Identifies medication pattern Real-time monitoring Identifies partial adherence	Expansive Technical support required Inconvenient for the patient Overestimation with improper use Pressure on patients	[81,82,83,84,85,86,87,88]

DIRECT METHODS					

Direct observed therapy	Yes	No	Easy to apply Secure medication intake Results available in a short period	Possible hiding of tablets in mouth Ethical issues Cannot provide info about degree of nonadherence Mostly utilized in conditions when medication is taken over a specified period Potentially expensive in long-term perspective	[89,90,91,92,93]

Therapeutic drug monitoring using plasma and urine samples	Yes	Yes	High sensitivity High specificity	Expansive and intrusive Provides information about total and partial screening Single-point evaluation of adherence Needs expertise Subject to white coat adherence	[88,94,95,96,97,98,99,100,101]

Therapeutic drug monitoring with dry blood spot	Yes	No	Convenient Accurate Easy and quick	Susceptible to abuse Single-point assessment of adherence Individualized variations in metabolism Occurrence of white cost-effect Various other contributing factors to nonadherence No pattern of adherence Need technicians and professionals Not suitable for multidrug regimens	[90,93]


### 2.1. Indirect methods

Indirect methods for assessment of medication adherence to antihypertensive medications include interviews, questionnaires, pill counting, and electronic monitoring.

#### 2.1.1. Patient interviews

The first method that physicians use to confirm adherence to the treatment is interviewing patients about their way of managing medications and the frequency of missing the medications in daily life. This method is surely the simplest approach, but various studies [25,26] have reported that interviewing a patient is like tossing a coin. Durand et al. [27] conducted a qualitative study to investigate various factors elucidating medication adherence among patients with treatment-resistant hypertension. Their results demonstrated the difficulty of distinguishing patients with good adherence versus patients with poor adherence with the interview/conversation method. Very few studies in Africa [28,29,30] had utilized interviews to investigate various perceptions, factors, and beliefs affecting nonadherence to medications; however, the utilization of interviews as a method of measuring medication adherence cannot be demonstrated based on the available studies. A descriptive cross-sectional study was done by Adebolu and Naidoo [31] in KwaZulu-Natal in South Africa. Although 95% of the hypertensive patients self-reported a good adherence to medication, effective BP control was still not observed among them. It was concluded that medication adherence was overreported, and an objective method of measuring adherence rate is urgently needed in the community.

Various studies have consistently reported that interviews provide overestimated information about patients’ adherence to the treatment [26,32,33]. Patients tend to provide incorrect information about the periods of nonadherence either intentionally or unintentionally to please the clinicians or to avoid time-consuming discussions. Another point to consider is the intrinsic nature of adherence, which is highly a variable and dynamic process, so it becomes difficult to specifically identify a patient who will remain adherent to the treatment at one stage and will be a poor adherent during others [34]. Indeed, questioning patients could be one of the easiest and cheapest methods for the assessment of adherence in clinical settings, provided patients honestly answer all the questions and admit the occasional presence of suboptimal adherence. However, these methods are greatly biased and often provide limited and irrelevant information about taking medication.

Taken together, it is agreed that these methods cannot be utilized for the assessment of adherence to the medications. However, in a large study conducted in eastern European countries (Austria, Hungary, and Slovakia), it was concluded that information about adherence to the treatment can be better obtained in a structured interview using a questionnaire with simple questions [35].

#### 2.1.2. Questionnaires

Questionnaires highlighting various issues of treatment adherence are considered qualitative and subjective measures of assessment. Generally, these questionnaires are completed by patients with the assistance of health care professionals [36]. This is one of the most used methods in clinical settings. More than 600 studies have assessed adherence to antihypertensive medications using a questionnaire. Questionnaires and self-adherence scales are available in various languages; almost 43 scales are written in English; however, few are mainly utilized in the field of hypertension and cardiovascular diseases. The main questionnaires used in hypertension treatment are the Morisky, Green, Levine (MGL) [37], the Morisky Medication Adherence Scale (MMAS) [37], the Hill-Bone Compliance to High Blood Pressure Therapy Scale (HBCHTS) [38], the adherence self-report questionnaire [39], the stages of change (SOC) for adherence measure [40], the Brief Medication Questionnaire (BMQ) [41] (Table 1). The validity and reliability of these questionnaires were reviewed by Perez-Escamilla et al. [36] and further discussed comprehensively in the review by Culig and Leppee [42]. The Cronbach’s α was utilized to determine the internal consistency as measure of reliability in each validation.

The most well-known and commonly used methods is the medication adherence questionnaire developed by Morisky et al. [37]. For the first time, in 1986, this scale was evaluated among hypertensive patients. This questionnaire is short and provides information about patients’ behavior in taking medication, frequency of missing medicine because of forgetfulness, and barriers to treatment adherence. It has been reported that the Morisky scale overestimates medication adherence as compared to the Medication Event Monitoring System (MEMS) [43]. In various studies [44,45,46,47,48,49] conducted on the African population, the questionnaire remains the predominant method of measuring medication adherence. In a recent paper, de Terline et al. [50] conducted a cross-sectional study in 12 sub-Saharan African countries to assess adherence to salt restriction and medication among patients with hypertension. They utilized the eight-item Morisky modified adherence scale [51] for the assessment of medication adherence and highlighted that a considerably high proportion of hypertensive patients (64.4%) were poorly adherent to the medication. This figure was in line with the findings reported by Abegaz et al. [52], who concluded that the prevalence of nonadherence to medication among hypertensives in Africa was 62.5%. However, their study may not have provided a comprehensive picture of medication adherence because included studies utilized a single tool for the assessment of antihypertensive medication adherence.

Various studies performed in this region utilized the eight-item Morisky modified adherence scale for the assessment of medication adherence among hypertensive patients [53,54,55,56,57]. These studies reported that just 8%–50% of patients were adherent to medications. However, one of the limitations highlighted in these studies was the introduction of recall bias because of the self-reported questionnaires, as patients were not assessed for their cognitive ability. Therefore, it may not have provided a true picture of the adherence levels among patients.

The HBCHTS was specifically developed for patients attending primary health care settings in South Africa [58]. The study was conducted by Lambert et al. [58] among the high-risk population and demonstrated a low compliance rate and poor BP control. This questionnaire is not only meant for the assessment of medication adherence but also can be utilized to get information about adherence to dietary salt intake and appointments. In comparison to the Morisky questionnaire, the HBCHTS provides detailed information about the compliance behavior of patients; however, the ability to identify medication adherence was inconsistent with both the questionnaires [58,59]. Therefore, neither of these questionnaires is recommended for clinical use. An adapted Turkish version with high construct validity and internal consistency is considered more reliable [60]. The Turkish HBCHTS scale also includes questions inquiring about risks and potential reasons for noncompliance, which may not only help researchers to identify personal factors contributing to noncompliance but also help health system–related problems. Nashilongo et al. [44] conducted a study in Namibia to validate the HBCHTS for the assessment of adherence to antihypertensive medication. They preferred the HBCHTS due to additional items for the assessment of adherence. It was concluded in their study that a modified version of the HBCHTS is reliable for the assessment of adherence to antihypertensive medications.

Although there is a limited performance of available self-reported questionnaires, it is important to highlight their advantages and disadvantages. The main advantage of using a questionnaire is that they are relatively simple and cost-effective and can easily be repeated [11]. However, these subjective approaches have been criticized because of frequently inaccurate findings, overestimated drug adherence, and involvement of biased results due to patient behavior [61,62]. But still, they are worth existing and highlight the role of investigating the adherence to medications. They also have an educative value and play a role in initiating a conversation with patients about medication adherence [63].

#### 2.1.3. Pill counting

Counting the number of consumed pills is one of the simplest and most cost-effective methods implemented in clinical settings [102]. In this indirect method of measuring adherence, several consumed dosage units are counted between two scheduled appointments with the doctor, followed by a calculation of the adherence ratio by comparison with the total units received by the patient [32,102].

Porter et al. [73] investigated the effectiveness of pillbox clinics to improve BP and measure medication adherence. They reported a clinically significant reduction in BP with a pillbox trial that was supervised by a pharmacist. Most studies have pharmacists; however, in their study, they propose that health care providers assist with pillbox filling, which may improve overall cost efficiency. Various studies have reported that pill counting can be used for the assessment of medication adherence [74,75,76]. In a study conducted [103] on 200 patients with mild to moderate hypertension in the Netherlands, it was reported that pill counting could be useful to measure medication adherence. Later, Perseguer-Torregrosa et al. [80] used three separate methods (pill counting, as the gold standard method, and Haynes-Sacket Method and Morisky-Green questionnaires) for measuring medication adherence, and the estimated medication nonadherence was reported to be 62.8% with the pill counting method as compared to 3.1% and 36%, respectively, with other two methods.

Pill counting is also utilized for measuring adherence in the African region. Adeyemo et al. [77] conducted a treatment adherence trial among 544 individuals with untreated hypertension; medication adherence was investigated with the pill counting method, and high medication adherence showed a better BP control. El Zubier et al. [78] did a cross-sectional study on hypertensive patients, and medication compliance was estimated with pill counting and self-reporting. Compliance of 60% was measured with pill counting methods. Previously, Maro and Lwakatare et al. [79] also estimated medication compliance among Tanzanian hypertensive patients with pill counting, and they concluded that medication adherence is related to frequency, time of dosing, and the number of drugs.

Although the pill counting method is easy and cost-effective, it has several limitations; for example, patients can mislead physicians by throwing away the pills before visiting the clinic. Although this method can be utilized for various formulations, such as tablets, capsules, etc., this method may not be appropriate for medications with nondiscrete dosages. Furthermore, the chance of having surplus medication is completely ignored, as it is a common practice among patients to refill the bottle before the medication is finished [32]. Additionally, an arbitrary cutoff value is used to differentiate between adherence and nonadherence, which further led to discrepancies in the determination and comparison of medication adherence among different studies [102]. Apart from this, the pill counting method is unable to characterize the adherence pattern and its causes. Furthermore, Rudd et al. [104] also reported on high pill count variability among subjects and commented that pill counting may be an unreliable method for measuring medication adherence. Therefore, this method may not provide accurate information about medication adherence [61,105,106]. However, electronic monitoring is an alternative approach to overcome this limitation. With electronic monitoring, pills are counted automatically with a device at the patient’s home [102].

#### 2.1.4. Electronic monitoring

The Medication Event Monitoring System (MEMS) was the first device that was commercially available for the measurement of adherence in clinical trials. MEMS is based on the principle of incorporating a microcircuitry device into pharmaceutical packages in such a way that the skill required to open the drug package can be analyzed and communicated in real time. MEMS was originally designed as an electronic cap for recording every event of opening and closing a standard medication bottle. This technology for measuring and monitoring medication adherence, such as electronic pill bottles, had been used in the past [81,107]. A comprehensive range of electronic systems with automated pillboxes is commercially available. Interesting continuous data can be obtained utilizing these devices, highlighting between and within-subject variability medication intake behavior along with changing nature of poor adherence [82]. Various studies have demonstrated that adherence measurement with MEMS is highly accurate, with 97% concordance between assessed adherence and plasma concentrations of the drug [85,108], and it provides in-depth information about individualized drug-taking behaviors and can be a gold standard for the measurement of adherence and the given feedback has resulted in improving medication adherence after being integrated into care systems. Additionally, patients can also be given a reminder in real time about the dosage, which is taken, and missed doses.

Burnier et al. [84] evaluated the potential benefits of electronic monitoring of drug compliance among treatment-resistant hypertensive patients. They assessed that 50% of the patients who were presumed to be resistant to the treatment were not taking their medications. Later, Wetzels et al. [87] designed a study to evaluate the effectiveness of electronic monitoring in measuring treatment adherence and BP regulation. They supported the assumption that poor adherence plays a critical role in treatment-resistant hypertension. They also provided evidence that electronic monitoring of medications is effective in measuring adherence to the treatment and results in the normalization of BP. Their findings agreed with other studies by Leenen et al. [88,98].

However, contrary findings were obtained in a study performed by Mehta et al. [99]. They compared electronic pill bottles and bidirectional text messaging about their impact on improving medication adherence on BP control among patients with uncontrolled hypertension. Medication adherence was measured as the number of days that the patient opened the pill bottle or that a response was received by text message. Their results showed a high proportion of medication adherence; however, there was not a significant improvement in BP control. They further suggested that providing electronic pill bottles or bidirectional text messaging with feedback to patients may not be enough in improving BP. It was further explained in their study that reasons for not showing any improvement in the control of BP could be that nonadherence to the medications was not the primary reason of low BP control in the studied population. Despite adherence to the medications, patients also need to escalate the dosage of the medication or to take additional drugs in case of consistently elevated BP. Furthermore, considering that medication adherence was higher in both the intervention arms, patients might need to have even more adherence to the medication. Also, opening a pill bottle or responding to the text messages may not be the actual reflection of medication taken; it may have been done to avoid the negative feedback. Furthermore, the primary outcome was based on a just one follow-up reading of the BP. Other studies also showed a poor correlation between levels of adherence and BP control [109,110]. This weak correlation could also be because adherent patients are not properly treated regarding dosage and type of drug.

These studies were performed in various parts of the world, including developed and developing countries; however, only a few studies reported on measuring medication adherence to antihypertensive treatment with electronic monitoring in the African region. Bovet et al. [111] performed a study in Seychelles to monitor compliance to antihypertensive medication using electronic pill containers. It was concluded that compliance to antihypertensive medication was quite low in the studied population. Later, a comparative study was performed by Ayoade and Oladipo [112], who investigated the correlation between a self-report questionnaire and electronic monitoring of the medication adherence assessment. A very weak correlation between the two methods was observed; therefore, both methods are not equivalent nor interchangeable for the assessment of medication adherence.

Despite the abovementioned benefits of electronic monitoring of medication adherence, the main challenge with MEMS is its implementation in large clinical settings. The electronic monitoring of package entry could be an indirect measure of the taken dose because there is a chance that the package is activated, but the dose is not taken. Some of these approaches are often expansive and time-consuming for health care practitioners and susceptible to misinterpretation by patients; therefore, this method has not been considered very effective [86]. The first issue is the assumption that the patient forgot to take medication; however, there are various other factors responsible for nonadherence [113]. Furthermore, providing feedback about missed doses could be intrusive for some patients, causing stigma, and may lead to treatment discontinuation. Therefore, various technical, legal, and ethical issues need to be considered.

All abovementioned indirect methods can be easily implemented in clinical settings. Their accuracy is, however, very limited. Conversely, direct methods involving direct observation of medication or monitoring of the drug or its metabolite levels in body fluids may provide more reliable information about medication adherence among hypertensive patients.

### 2.2. Direct methods

Direct methods include directly observed therapy and therapeutic monitoring of drugs and various body fluids. These are summarized in Table 2.

#### 2.2.1. Direct observed therapy

In the directly observed therapy (DOT) method, the patient’s act of consuming medication is directly supervised by someone who is not a family member. This method has been considered a standard method of care for the treatment of tuberculosis (TB) in developing countries and certain settings in developed countries [114]. This approach has emerged as the most accurate method and is suggested to be the closest one to the gold standard for measuring medication adherence [115]. In a study conducted by Hameed et al. [93], data were collected from treatment-resistant patients who attended a DOT clinic. Their results showed that only 50% of enrolled resistant hypertensive patients had truly resistant hypertension, while the other half of the patients were nonadherent to the antihypertensive medication. Recently, Ruzicka et al. [90] performed a prospective observational cohort study among hypertensive patients. Only patients with complete concordance with pharmacy records, pill count, and treatment regimen were enrolled in DOT for a one-month follow-up. They reported that the nonadherent percentage was high among patients who were categorized as adherent to the treatment as per questionnaires, pristine refill records, and correct pill counts. Their results concluded that rigorous methods such as DOT should be utilized for the medication adherence measurement among treatment-resistant hypertensive patients. Similar findings were documented by Polonia et al. [116]. Recently, Pio-Abreu et al. [92] investigated the impact of DOT for evaluating not only adherence and diagnosis of resistant and refractory hypertension but also on BP control. Their results revealed that DOT at the hospital not only helps to confirm the diagnosis of hypertension but also results in better BP control.

The main advantage of giving medication under direct observation is that the resultant effect of medication on BP can quickly be apparent, including side effects, if any. However, this method can only be utilized for medical conditions where medication should be taken over a specified period, but for hypertension, there is no finite duration of medication that makes DOT unsuitable for treating hypertension [91]. Furthermore, patients may also hide medications in their mouth and discard them later. Additionally, this method also involves ethical issues and cost components. Furthermore, it requires patients to attend the clinic and be supervised by senior staff. Additionally, this method is greatly dichotomous to identify adherence versus nonadherence, hence it cannot provide information about the degree of nonadherence (partial versus complete), which can be possible with TDM.

#### 2.2.2. Therapeutic drug monitoring utilizing plasma and urine samples

Therapeutic drug monitoring is a pharmacokinetically guided drug therapy for the assurance of exposure to a drug being utilized for treatment effectiveness and toxicity. In this method, the steady-state concentration of medication is measured in plasma and relative to a standard concentration at a specific time point. Various pharmacodynamic markers can also be measured to investigate direct medication adherence. Clinical and physiological markers include pharmacodynamic markers of exposure after a given antihypertensive treatment, such as bradycardia in patients on beta blockers, hyperuricemia, or gout in patients on diuretics; high plasma renin concentration in patients on diuretics or renin-angiotensin system blockers; or rise in urine N-acetyl-seryl-aspartyl-lysyl-proline (AcSDKP) concentration in patients on angiotensin-converting enzyme (ACE) inhibitors [93]. Pharmacological screening may also provide information about partial or total adherence, depending on the actual detection of antihypertensive drugs (AHT) in comparison to the prescribed drugs. The most recent guidelines given by International Society of Hypertension emphasizes the screening of nonadherence utilizing drug monitoring in blood or urine [117,118].

Various clinical laboratories use liquid chromatography coupled with tandem mass spectrometry (LC-MS/MS) for the detection of antihypertensive drugs in body fluids. Among various body fluids, blood and urine have extensively been explored.

Several studies [94,95,96] have utilized blood samples for TDM and reported a great variation in the percentage of patients (19%–86%) who were nonadherent to antihypertensive medications. The first time, Ceral et al. [94] performed a serum drug analysis on 84 patients with uncontrolled arterial hypertension. They reported that serum drug levels were found in the sera of 34.5% of patients, while 65.5% were diagnosed as nonadherent to the medications. This study was followed by Štrauch et al. [95], who performed a study on a bigger group of patients and analyzed that noncompliance with antihypertensive treatment among resistant hypertensive patients is very high (47%). Similar findings were observed by Florczak et al. [96], with a nonadherence percentage of 86% among treatment-resistant hypertensive patients; however, the small sample size was one of their limitations. Additionally, various analytical methods using plasma for the determination of adherence have been adapted to multiparametric settings [97]. Utilizing this method, the frequency of nonadherent patients was reported to vary between 42% and 86% [119,120,121].

Furthermore, toxicological urine screenings have also been used for the assessment of drug intake in cases of apparent resistant hypertensive patients. Chemical adherence testing using a urine sample is mostly a qualitative measure [122]. In the past, toxicological urine screening used to be performed with gas chromatography–mass spectrometry (GC-MS); however, for the last decade, the improvement of liquid chromatography–mass spectrometry (LC-MS) had led to the development of analytical procedures for the determination of various drug levels, including antihypertensive drugs [123,124,125,126]. A significant number of molecules can be accessed with the LC-MS method if a drug/any metabolite is excreted in the urine in a significant amount [97]. The method of toxicological urine screening was implemented by Jung et al. [100] on resistant hypertensive patients in a clinical setting for the assessment of adherence, and they reported that 70% of included patients had incomplete adherence. It was concluded in their study that this method can be utilized for the detection of low adherence. Gupta et al. [101] postulated that nonadherent hypertensive patients had an improvement in adherence to antihypertensive medications after having been assessed with LC-MS–based biochemical analysis. The spot urine screening has been performed in various resistant hypertensive patients included in clinical trials, and nonadherence rates have been reported to vary between 48% and 76% [127]. It has been stated that urinary adherence screenings are quite feasible in outpatient consultations and are promising in amplifying adherence levels [101]. Recently, Lane et al. [118] recommended the use of urine or blood samples for measuring the drug levels.

Urine samples are preferred for their inexpensive collection and noninvasive nature as compared to blood samples; furthermore, urine sampling is easy to implement, and samples can be stored at room temperature for a few hours and require simple analytical procedures. However, In a study done by Ritscher et al [128], the quantitative serum analysis method was compared to qualitative urine assays, and adherence rates were assessed among hypertensive individuals. The results demonstrated that drugs with low bioavailability, reduced renal excretion, or high metabolic rate may account for underestimation of adherence via urine analysis. Furthermore, concentrations of the drugs in the urine do not reflect the actual time when the drug was administered due to variation in hydration, pH, and frequency of urination [118]. They further suggested that evaluation of serum concentration could be a better approach in targeting active drugs and for adherence assessment. The most used matrices for TDM of antihypertensive drugs are plasma and urine [129,130,131]. However, saliva is another biological matrix with easy collection and storage. The collection of saliva is noninvasive and does not require any technical expertise. Therefore, multiple sampling over a period is possible and may also limit the presence of white coat adherence. Avataneo et al. [131] tested 14 antihypertensive drugs simultaneously in plasma, urine, and saliva. TDM was evaluated using ultra-high-performance liquid chromatography coupled to tandem mass spectrometry (UHPLC-MS/MS). The results obtained from salivary concentrations relative to plasma and urine analysis were accurate, with overall sensibility and specificity of 98% and 98.1%, respectively. However, there are various factors, including molecular mass, lipophilicity, ionization state, protein binding, water consumption, time from dosing, and salivary pH that could contribute to variable drug concentrations in the oral fluids and interpatient variability.

Measurements of drug levels in biological fluids at a one-time point may not provide substantial information about the duration of nonadherence; that issue, however, can be addressed with the self-collected dry blood spot (DBS) method at various intervals [132,133]. The DBS is reported as a convenient and accurate method for the measurement of nonadherence in clinical settings [121]. It is a patient-friendly technique and can easily be performed by a single prick; it enables easy and quick sample collection in case nonadherence is suspected and minimizes the risk of nonadherence [134,135,136]. However, there is a subtle difference between plasma and dry spot drug monitoring. The whole blood is used in the dry spot method as compared to plasma, and drugs showing adherence to red blood cells can have a better advantage over the dry spot method. In addition, various parameters, such as blood viscosity, hematocrit, and the shape of the drop, should be taken into consideration for reliable measurements of drug concentrations.

Peeters et al. [121] performed TDM utilizing DBS for the quantification of eight different antihypertensive drugs. Various drugs included in their study were ACE inhibitors; enalapril (enalaprilate) and perindopril (perindoprilate), angiotensin II receptor blockers (ARB); losartan (losartan carboxylic acid [ca]) and valsartan, diuretics; hydrochlorothiazide and spironolactone (canrenone), calcium channel blockers; amlodipine; and nifedipine. Analytical validation was performed using UHPLC-MS/MS. To compare quantitatively, samples for DBS and plasma levels were collected simultaneously and analyzed with Deming regression and Bland-Altman analyses. It was concluded that DBS is a reliable method for measuring medication adherence. These findings were also corroborated with results of other studies [132,137] reporting that because of easy sample collection, storage, and transport, the DBS method can be a better option in resource-limited settings.

TDM is also employed for the evaluation of adherence among patients with chronic diseases on the African continent. Recently, Adewuya et al. [138] investigated the use of serum uric acid levels as a marker for predicting medication adherence. In their study, they utilized both the MMAS-8 scale and levels of uric acids among hypertensive patients and concluded that there was a linear relationship between higher levels of uric acid and the percentage of increasing nonadherence to the medications; therefore, serum markers can be used as a measure of medication adherence. In 2017, Jones et al. [139] measured amlodipine and ACE inhibitor concentrations among hypertensive individuals utilizing an LC-MS assay. The main findings of the study were that 20% of patients had an undetectable amlodipine concentration, and 27% had unsuppressed ACE; this was associated with significantly higher BPs compared with those with steady-state concentrations or suppressed ACE. Additionally, they did not report any correlation between adherence levels measured with pill counting, dosing schedule, etc., to TDM. They further suggested that this tool should be routinely used for evaluating medication adherence to optimize medication concentration among patients with treatment-resistant hypertension.

This method has high sensitivity and specificity, but the involvement of high cost makes its use limited in clinical settings. These methods are susceptible to abuse (mixing of medications in urine samples), single-point assessment of adherence, individualized variations in metabolism, and the occurrence of white coat adherence when patients are aware of the schedule of tests. Furthermore, this method has limited utility in measuring long-term adherence, and it can only measure the plasma concentration of drugs with a long half-life.

Measurement of biological markers in body fluids is objective, but it is often expansive and intrusive because of the requirement of the collection of body specimens. Various other factors could also contribute to the nondetection of a drug in urine, such as pharmacokinetic properties of the drug that can be strongly influenced by food, genetic factors, and drug-drug interactions. Similarly, detection of a significant number of drugs in plasma can not confirm adherence to the treatment because of the possibility of the white coat adherence phenomenon. Despite the mentioned limitations with these measurements, nondetection of drugs in body fluids is often associated with nonadherence to the treatment. In a nutshell, these measurements may just provide a brief picture of the adherence phenomenon, which is a dynamic process and can vary at any time, and are not suitable for long-term monitoring of adherence to prescribed drugs.

## 3. Hair Analysis as a Potentially Effective Method to Measure Adherence to Hypertension Treatment

To overcome the abovementioned limitations of TDM in body fluids, hair analysis has been proposed for the measurement of medication adherence. This method has mainly been used in forensic studies to unveil drug history of an addicted person and in solving crime cases [18,19,21,22,140]. Drug incorporation into hair is a complex process, and the exact mechanism is not known; however, it is suggested that the bloodstream is the main route for delivering drugs to hair follicles, which can also get contaminated via drugs from external sources, sweat, and sebum [141,142]. Various studies [143,144] have analyzed hair, an easily available and stored matrix, for determining the concentrations of antiviral therapies and drug-resistant TB medications [145]. Measurement of drugs in hair may also provide information on past exposure and history of medication use. Besides its noninvasive nature, using hair to test for drugs provides a major benefit, as it offers a broader observation window in comparison to testing urine or blood [146]. On average, hair growth can take weeks to months, thus allowing enough sampling period to investigate adherence to prescribed medication over time.

Importantly, different compositions of amino acids in hair can make it possible to estimate or measure varied functional groups in different types of hair. Similarly, advanced characterization and classification techniques, such as LC-MS and GC-MS, can be applied to detect different drug metabolites, like amphetamines and opiates, in response to drug use [22]. As such, there has been a growing interest in using hair analysis as an effective method to monitor patient drug adherence. Several clinical studies have been published on the topic. For example, Ferrari et al. [147] found an excellent correlation between hair drug composition and self-reported drug use; they even showed a substantial relationship between the dose used and hair drug level for headache treatments, such as amitriptyline, citalopram, delorazepam, and duloxetine. Others [148] have shown that human hair indeed integrates diverse compounds, including antiretroviral medications such as lopinavir and ritonavir, as it grows, even equating these hair drug levels to those found in plasma, further indicating that this method can also be applied to monitor adherence to human immunodeficiency virus (HIV) medication. Extensive literature is available on the utilization of hair samples for measuring antiretroviral levels in hair samples for measuring adherence and exposure [20,149,150,151,152]. In addition, emerging research [145] even indicates that hair drug analysis is currently developed as an effective strategy for TDM during drug-resistant TB treatment.

Also, as a key component of monitoring BP, there has been growing interest in using hair analysis to monitor the use of hypertensive drugs. Interestingly, the hypertensive drug minoxidil, which was initially introduced to treat high BP in the 1970s [153,154], is long known to be detected in hair follicles. Since then, accumulating research indicates that hair analysis can be effectively used to detect the concentration of minerals, such as calcium and magnesium, in hypertensive patients to inform on their nutrition status [155,156]. Hair analysis has also been utilized for the determination of cardiovascular drugs in the context of doping [157,158]. Later, Lo Faro et al. [159] reported on the successful use of the UHPLC-MS/MS method for quantifying diuretics in hair, including acetazolamide, brinzolamide, dorzolamide, and their metabolites. Recently, Taus et al. [24] described and validated the possibility of using UHPLC-MS/MS for the quantitative measurement of beta blockers (metoprolol, sotalol, labetalol, atenolol, nebivolol, bisoprolol, and nadolol) and calcium channel blockers (lercanidipine and amlodipine) among hypertensive subjects. The study was performed on n = 34 hair samples collected from patients undergoing long-term antihypertensive therapy. The practical applicability of UHPLC-MS/MS was evaluated according to international guidelines in terms of linearity, sensitivity, selectivity, and extraction recovery [160].

Overall, although there is certainly a scarcity of evidence affirming the use of hair analysis to monitor patient adherence to drugs taken for BP, hypertensive agents such as acetazolamide and brinzolamide have been shown to accumulate in the hair after being taken orally [159], further indicating the importance of developing this method to monitor medication adherence, especially in a resource-poor setting like Africa, where there are certainly reduced trends of adhering to prescribed medication [161].

Considering the cost factor in low-resource settings, minimum cost is involved with the collection, transportation, and storage of hair samples. Hair samples can be stored at room temperatures without any biohazardous precautions [162]. Additionally, hair collection is noninvasive, and there is no need for sterile equipment or technical skills. Although, the equipment required for hair analysis using LC-MS/MS is expensive, Gandhi et al. [163] developed a cost-effective method for the detection of antiviral drugs in hair using thin-layer chromatography. Further studies are mandatory to utilize this cost-effective method as a monitoring tool for assessing medication adherence in low-resource settings. Future work to develop more low-cost hair analysis methods and their dissemination for monitoring of antihypertensive medication adherence in resource-limited settings is warranted.

Furthermore, Kholi-Lynch et al. (2022) recently reported that a significant proportion of the public health care budget is spent on diagnosis, treatment, and management of hypertension [164]. The economic burden due to hypertension is even higher due to the loss of economic productivity. Considering the long-term benefits of adherence screening in the prevention of myocardial infarctions and stroke events [165], and reduced cost involved with collection, storage, and transportation of hair samples, mass spectrometry–based hair analysis will still be a cost-effective strategy and will be a better alternative to other popular matrices, such as blood and urine, because it also provides retrospective analysis [166].

Although, there is a substantial literature about the validity of quantitative results obtained from hair analysis, the results should be interpreted with caution because of some unsolved questions, such as the contribution of external contamination or cosmetic treatment and role of genetic factors to drug deposition in hair [167,168]. The use of cosmetic products that include strong bases may reduce the drug contents and stability of the drugs. The association between hair pigmentation and the concentration of accumulated drug has also been revealed; binding of melanin could account for that [169]. Specifically for basic drugs, dark hair with high melanin content may bind a higher quantity of drugs than light-colored hair [170]. Furthermore, interindividual and interdrug variation have also been observed in the hair contents of antiretroviral drugs [168]. All these factors should be considered when the concentration of any drug in hair is utilized as a measure of long-term adherence to medication.

## 4. Conclusion

Nonadherence to treatment is highly prevalent and a major issue in the management of patients with uncontrolled hypertension. The accurate evaluation of medication adherence is of crucial importance for developing interventions for reducing nonadherence, controlling hypertension, and preventing drug-related complications. However, measuring treatment adherence has often been challenging due to the subjective nature of assessment methods and their limited use in clinical settings. The identification of accurate and clinically feasible methods for estimating medication adherence is imperative for the control of BP and reducing cardiovascular-related morbidity. This review provides health care professionals a summary of the currently used methods (direct and indirect) for the assessment of adherence to antihypertensive therapies. Further, it has also highlighted various studies performed in various parts of the world, but limited studies are available on measuring medication adherence to antihypertensive treatment within the African region.

Considering the strengths and limitations of various indirect and direct methods of biochemical drug screening, particularly of hair samples, should be embedded in the routine management of hypertension in the future. This method has shown to be useful in clinical practice for the measurement of adherence to antiviral drugs and, recently, has also been demonstrated to have the potential for high-throughput screening of various other drugs. However, future studies are required to validate the potential of hair analysis as a method to monitor medication adherence in hypertension treatment.
